# Molecular identification and phylogenetic analysis of the mitogenome of *Solenaia oleivora* MG

**DOI:** 10.1080/23802359.2020.1788435

**Published:** 2020-07-14

**Authors:** Pengyu Chen, Danni Li, Xuxu Chen, Genfang Zhang, Shoubao Yang

**Affiliations:** a College of Life Sciences, Shaoxing University, Shaoxing, P. R. China; b School of Agriculture and Bioengineering, Jinhua Polytechnic, Zhejiang, P. R. China

**Keywords:** *Solenaia oleivora* MG, mitogenome, molecular identification, phylogenetic analysis

## Abstract

*Solenaia oleivora*, belongs to Bivalvia, Unionidae, and Gonideinae, is a burrowing bivalve uniquely distributed in China. In this study, the complete mitochondrial genome of *S. oleivora* MG was sequenced and determined. The complete mitogenome of *S. oleivora* MG is 16,392 bp in total length, consist of 22 *tRNA* genes, 13 protein-coding genes (PCGs), and 2 *rRNA* genes. The overall base composition of the *S. oleivora* MG mitogenome is 36.90% A, 23.85% T, 27.09% C, and 12.16% G, respectively, exhibits a similar AT bias (60.75%) feature to other invertebrate bivalve mitogenomes. The phylogenetic analysis that *S. oleivora* MG clustered in genus Solenaia. This result provides useful data to the conservation and sustainable utilization of *S. oleivora* MG and other invertebrate mussels.

*Solenaia oleivora*, belongs to Bivalvia, Unionidae, Gonideinae, is a burrowing bivalve uniquely distributed in Hunan, Hubei, Jiangxi, Zhejiang, Jiangsu, Anhui, and Henan province of China (Xu et al. 2005, 2006, 2013; Li et al. 2012; Wang et al. 2015; Wu et al. 2018; Bolotov et al. 2019). It is an economically important freshwater mollusk with fast growth, large individuals, and high nutritional value (Xu et al. 2003, 2005, 2008; Yang et al. 2011). However, in recent years, its wild population declines rapidly because of water pollution and increasing capture pressure (Xu et al. 2005; Huang et al. 2015; Zhang et al. 2020). Identification of the complete mitochondrial genome, and make clear its phylogenetic relationships with other closely related species is necessary for the conservation and sustainable utilization of *S. oleivora* and other aquatic species (Tzeng et al. 1992; Liu and Cui 2009; Min and Park 2009; Chen et al. 2013; Huang et al. 2013; He et al. 2014; Wu et al. 2019).

The individual of *S. oleivora* MG was sampled from Chihe River, Mingguang city, Anhui Province of China (32°81′79.6′′N, 117°96′74.03′′E), and was kept in 99% ethanol in the Aquatic Service Platform of Shaoxing (accession no. SXAF20200219).

The complete mitochondrial genome of *S. oleivora* MG is 16,392bp in length, deposited in GenBank database with an accession number MT477834. It consists of 22 *tRNA* genes, 13 protein-coding genes (PCGs), and two *rRNA* genes. It is gene structure and arrangements are similar to the typical bivalve mitogenomes (Huang et al. 2013; Huang et al. 2015).

The total length of the protein-coding gene sequences is 11,118 bp. Except for the ND6 is encoded on the L-strand; all the other *PCD* genes (ND1–5 and ND4L, COXI-III, ATP6, ATP8, and CytB) are encoded on the H-strand. The total length of all *tRNA* genes is 1433 bp, varying from 61 bp (tRNA^Gly^) to 71 bp (tRNA^Ala^). The 12S *rRNA* gene (843 bp) and *16S rRNA* (1287 bp) gene are located between two *tRNA* genes (*tRNA^Arg^* and *tRNA^Leu^*), and are separated by *tRNA^Lys^*, *tRNA^Thr^*, and *tRNA^Tyr^* genes. The gene structure and arrangement of *S. oleivora* MG are very similar to other mollusks (Huang et al. 2013). The overall base composition of the *S. oleivora* MG mitogenome is 36.90% A, 23.85% T, 27.09% C, and 12.16% G, respectively, exhibits an obvious and similar AT bias (60.75%) feature to other invertebrate bivalve mitogenomes (Huang et al. 2013; Yang et al. 2015).

The phylogenetic tree was constructed using the neighbor-joining method. The results showed that *S. oleivora* MG is clustered with other Solenaia mussels including *S. oleivora* (Huang et al. 2015), *S. carinatus* (Huang et al. 2013), and *S. carinatus* (GenBank accession number: NC_039839) (Figure 1). While it showed distant kinship with other shellfishes like *Lamprotula caveata* (GenBank accession number: NC_030336) and *Alasmidonta varicosa* (GenBank accession number: NC_038155). This study provides useful data to the conservation and sustainable utilization of *S. oleivora* MG and other invertebrate bivalves.

**Figure 1.**
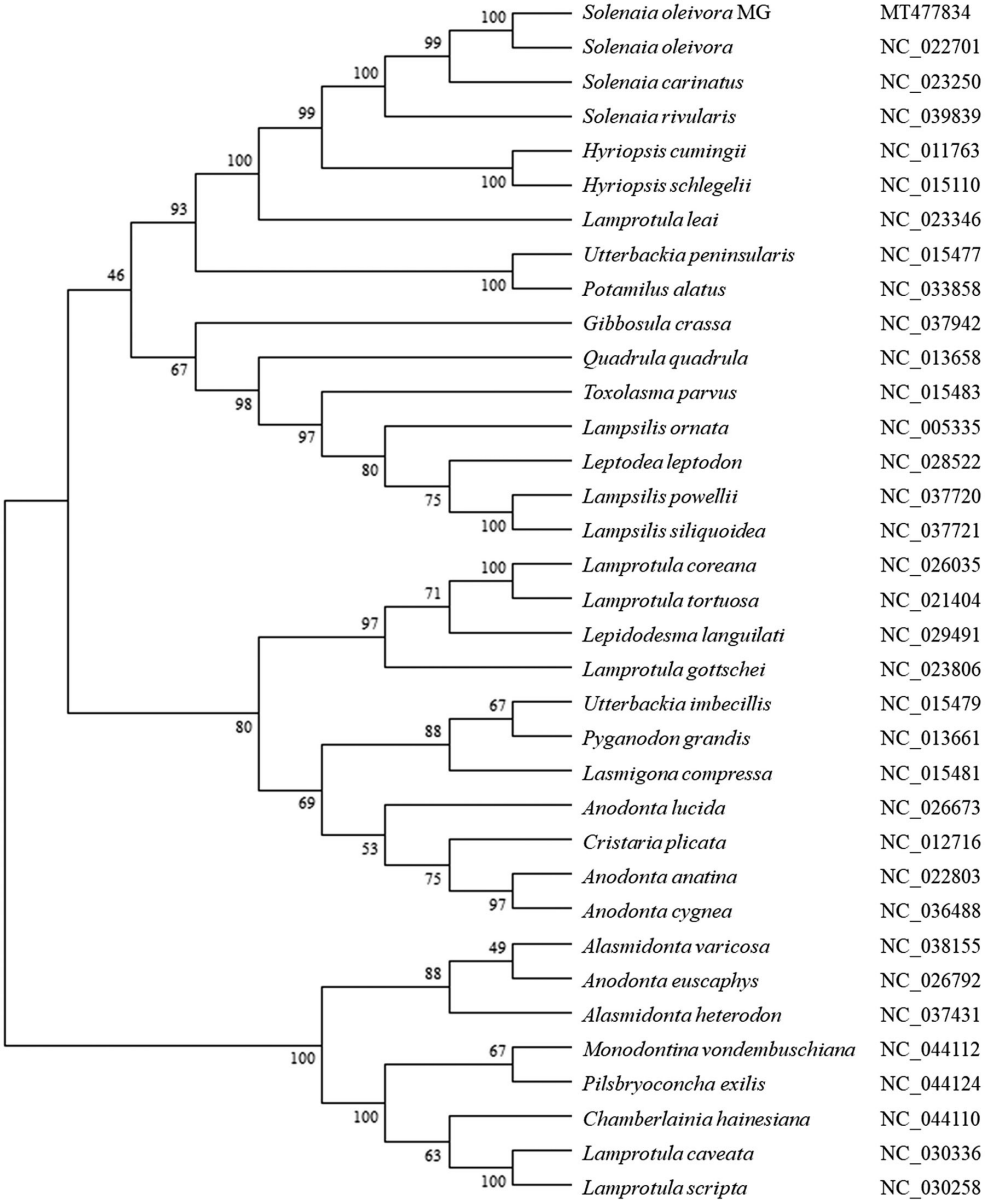
The phylogenetic analysis of *Solenaia oleivora* MG and other shellfishes based on the mitogenome sequences.

## Data Availability

The data that support the findings of this study are openly available at NCBI (https://www.ncbi.nlm.nih.gov), GenBank accession no. MT477834. And the data that support the findings of this study are also available from the corresponding author, Dr. Yang, upon reasonable request.
